# *Candida albicans* Goliath cells pioneer biofilm formation

**DOI:** 10.1128/mbio.03425-24

**Published:** 2025-08-08

**Authors:** Iana Kalinina, Duncan Wilson

**Affiliations:** 1 Medical Research Council Centre for Medical Mycology at the University of Exeter, Exeter, United Kingdom; Duke University Hospital, Durham, North Carolina, USA

**Keywords:** Goliath, adhesion, biofilm, hydrophobicity, host-pathogen interactions

## Abstract

*Candida albicans* is a fungal pathogen that can cause life-threatening bloodstream infections. Indwelling medical devices, such as catheters, play a critical role in their progression, as *C. albicans* can colonize these devices and form biofilms, which can then seed the bloodstream, resulting in recurrent candidemia. Under conditions of zinc restriction, *C. albicans* forms enlarged Goliath cells. Here, we show that Goliath cells exhibit enhanced adhesion to host tissue under static conditions, particularly to vaginal and oral epithelial cell monolayers. Moreover, Goliath cells are fully capable of forming hyphae and damaging host tissue. Notably, the adhesion of Goliath cells to abiotic surfaces surpasses their adhesion to epithelia or endothelia, suggesting that Goliath cells may possess a unique advantage in colonizing indwelling medical devices, such as catheters within the circulatory system. We also show that the cell wall of Goliath cells has increased hydrophobicity compared with yeasts. In microfluidic channels mimicking the shear stress present in the circulatory system, yeast cells exhibit negligible adhesion to plastic surfaces, even at minimal flow rates. In contrast, Goliath cells demonstrate robust adherence to abiotic surfaces, maintaining attachment even under supraphysiological shear stress conditions. We show that biofilms formed by Goliath cells are more metabolically active and structurally denser in terms of the volume of hyphae. A high number of adhesive “pioneer” Goliath cells in the base of the biofilm significantly affects their architecture and anchors them to abiotic surfaces. These insights into Goliath cells’ pathogenicity shed light on their potential role in colonization of medical devices.

**IMPORTANCE:**

Goliath cells, known for their large size and stickiness to plastic, are triggered by limited access to the essential mineral zinc. They are a specialized cell type of *Candida albicans*, a fungus that causes skin, oral, and vaginal yeast infections as well as severe bloodstream infections. Medical devices like catheters can make these bloodstream infections worse because *Candida* forms biofilms on them, which results in continuous seeding of fungal cells into the bloodstream. We found that Goliath cells adhere to vaginal and oral tissue better than normal yeast cells. Surprisingly, they stick even better to abiotic surfaces than to mammalian cells. This suggests they might have an advantage in attaching to catheters during *Candida* bloodstream infection. When we simulated blood flow, regular yeast cells did not stick to plastic surfaces, but Goliath cells attach even at very high flow rates. This allowed them to form much thicker and active biofilms on these surfaces. Understanding how Goliath cells work can help us figure out better ways to prevent and treat infections caused by *Candida albicans*, especially those related to medical devices like catheters.

## INTRODUCTION

The human commensal fungus *Candida albicans* is a prevalent member of the oral, gut, and vaginal microbiota (1, 2). Despite its normally commensal nature, *C. albicans* is also a common human pathogen, capable of causing a spectrum of infections ranging from mucosal and skin infections to life-threatening disseminated candidiasis (3). The mortality rates associated with systemic candidiasis are notably high, ranging from 19% to 40% and are influenced by the complexity of comorbidities that often accompany such infections (4, 5). Invasive candidiasis typically affects immunocompromised individuals, particularly those with severe illnesses in hospital settings (6). Invasive medical treatments, such as abdominal surgeries or trauma, allow *C. albicans* to breach gastrointestinal barriers, leading to systemic infections (7). The presence of indwelling medical devices, such as catheters, is also a major risk factor (5, 8–13). In the case of *C. albicans*, it is thought that translocation of yeast cells from the gastrointestinal tract to the bloodstream is responsible for catheter colonisation and subsequent biofilm formation (7, 14). With advancements in invasive medical procedures and the increasing life expectancy facilitated by improved medical care, there is a growing need to understand the mechanisms underlying bloodstream-disseminated candidiasis.

*C. albicans’* virulence is strongly related to its ability to alter morphologies in response to environmental cues (2). The yeast form is thought to be associated with commensalism and systemic dissemination, while hyphae are readily able to invade host tissue. This is facilitated by adhesins (including the Als family, Hwp1, and Hyr1) (8, 15).

As well as the yeast to hyphae morphological transition, *C. albicans* can form other cell types, including pseudohyphae and chlamydospores. Yeast cells are also able to undergo phenotypic switching. Our group recently discovered a novel *C. albicans* morphology – Goliath cells, the formation of which is triggered by limited zinc availability (16–18). Zinc is an essential micronutrient, and its availability to pathogenic microbes is actively restricted by the host in a process known as nutritional immunity (19). Unlike normal yeast cells, Goliath cells are substantially larger and have a remarkable adherence capacity to plastic surfaces under static conditions (18). Given the importance of medical devices in *Candida* infections, including dentures, joint prostheses, tracheostomy tubes, and catheters, the hyper-adhesive property of Goliath cells has considerable medical relevance. This paper investigated the pathogenic potential of Goliath cells and, specifically, whether they can adhere to abiotic surfaces under physiologically relevant shear stress, exploring their potential role as catheter colonizers.

## RESULTS

### Goliath cell formation and reversion

**Fig 1:**
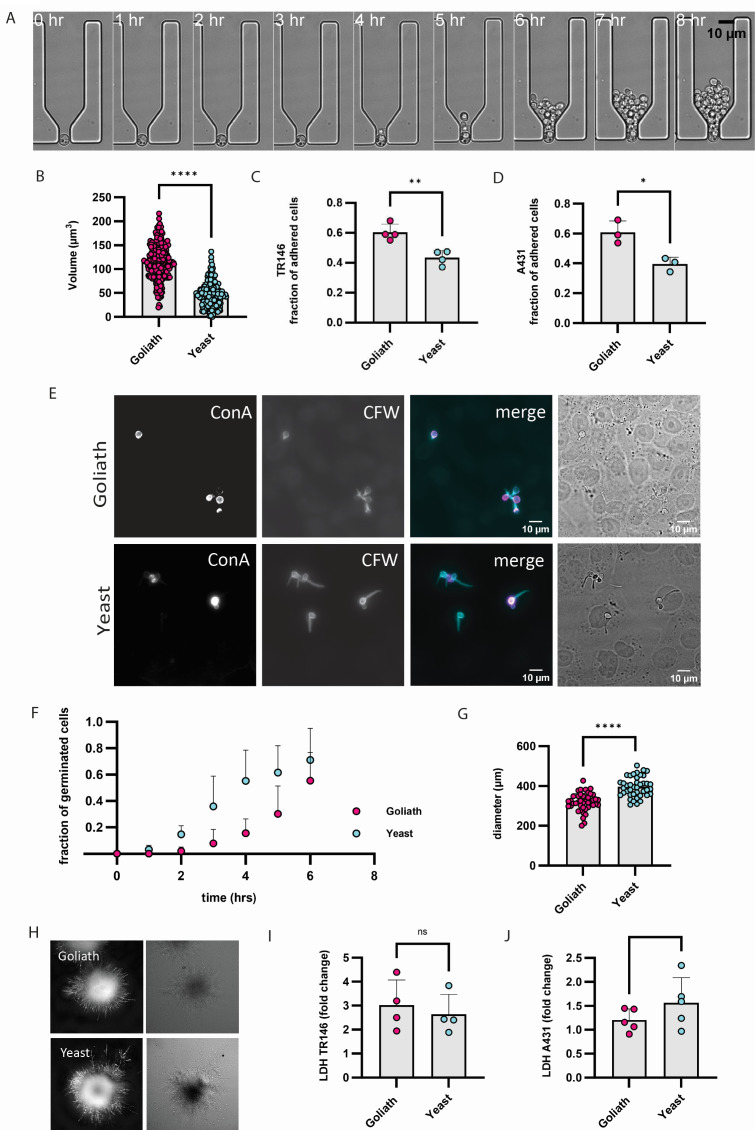
Goliath cell host-pathogen interaction. (**A**) Montage of time-lapse movie of Goliath cell trapped with microfluidic chip and perfused with replete YNB. Goliath cells produce daughter yeast cells upon exposure to zinc-replete medium under yeast condition (30°C, no CO_2_, YNB). (**B**) Volume of cells stained with FITC before inoculation and placed in YNB (yeast) and in YNB without zinc (Goliath) for 6 days (30°C, no CO_2_, shaking 180 rpm). (**C**) Adhesion to oral epithelia (TR146). (**D**) Adhesion to vaginal epithelia (A-431). Goliath or yeast cells were incubated on indicated epithelia for 15 min. Epithelia were then washed three times with PBS, and the percentage adhesion was determined by counting the number of CFU in the supernatant and in the washes. (**E**) Invasion of oral epithelia. Goliath or yeast cells were incubated on oral epithelia for 2 h. The samples were then fixed and extracellular fungi stained with Concanavalin A –Alexafluor 594 nm (first column and magenta). Epithelia were permeabilized with Triton-X100, and the entire fungal cells were then stained with Calcofluor White (second column, cyan). The third and fourth columns show the fluorescent channel overlay and the DIC, respectively. (**F**) Quantification of Goliath and yeast cell hyphal germination on oral epithelia over time. (**G**) Microcolony quantification. (**H**) Microcolony morphology. Individual Goliath or yeast cells were incubated on TR146 monolayers for 24 h, fixed, and stained with Calcofluor White. (**I**) Oral epithelial damage. (**J**) Vaginal epithelial damage. Goliath or yeast cells were used to infect TR146 or A-431 epithelia for 24 h and tissue damage was assessed by measuring the amount of lactate dehydrogenase (LDH) released into the supernatant. Statistics were performed using unpaired *t*-test. *P < 0.5; ****P < 0.0001.

Yeast and Goliath cells were generated by incubating yeast cells from a 24 h culture in minimal media (YNB, glucose) in low zinc media (YNB zinc dropout, glucose) either with (yeast) or without (Goliath) 25 µM zinc supplementation for 6 days at 30°C. A resultant Goliath cell is shown in the 0 h timepoint of Fig. 1A. To track the formation of daughter yeast cells, a microfluidics approach was taken (Fig. 1A). We designed a microfluidic chip to trap Goliath cells and collect the daughter cells produced by the mother cell. To achieve this, we generated a hydrodynamic trap with dimensions of 25 µm × 68 µm × 11 µm. Goliath cells were trapped between PDMS pillars spaced 6.75 µm apart from each other (chip design is shown in Fig. S1) and perfused with fresh media containing zinc to allow the formation of yeast cells. We observed that Goliath cells start to produce daughter yeast cells (Fig. 1A).

To further understand the dynamics of Goliath cell formation at a population level, we labeled yeast cells with fluorescein isothiocyanate (FITC) before inoculation into zinc-depleted medium. The cellular volume of inoculated (FITC-labeled) cells gradually increased over 5 days of incubation (Fig. S2). Daughter (unlabeled) cells produced during this incubation were comparable to the initially inoculated yeast cells. However, these yeast-sized daughter cells, themselves gradually enlarged over the course of the experiment (Fig. S2).

The final Goliath cell volumes are plotted in Fig. 1B alongside a yeast control in Fig. 1B. The median volume of Goliath cells was 116.4 µm^3^ compared with 46.08 µm^3^ for yeast cells.

### Goliath cells have increased adhesion to oral and vaginal epithelia

The ability of *Candida albicans* to change morphologies plays a critical role in pathogenicity (2). While the role of hyphal morphogenesis has been widely studied, the role of Goliath cells in host-pathogen interactions is currently unknown.

*C. albicans* causes vaginal candidiasis in otherwise healthy women as well as both oral and invasive, life-threatening infections in people with a weakened immune system (1, 20). In the case of superficial mucosal infections, the primary interaction between the pathogen and host involves adhesion of the fungus to host epithelia (21).

Both fungal cell types were then incubated on oral (TR146) or vaginal (A431) epithelial cells for 15 min, under hypha-inducing conditions 37°C, 5% CO_2_, the epithelia washed, and adhesion determined by counting the number of colony-forming units (CFUs) recovered in the supernatant and in each of 3 washes that followed. Compared with yeasts, Goliath cells exhibited significantly higher adhesion to both oral and vaginal epithelia (Fig. 1C and D; Fig. S3A and B). This suggests that the Goliath morphology may play a role in the initiation of mucosal candidiasis by anchoring the fungus to the host tissue more efficiently.

### Goliath cells exhibit delayed hyphal germination but cause similar ultimate epithelial damage

Having established that Goliath cells are more adherent to epithelial tissue, we next questioned whether this morphotype was suited for commensal colonisation or pathogenic potential. This is important because it is known that different types of *C. albicans* yeast cells respond differently to hypha-inducing environmental cues. For example, opaque cells do not form hyphae in response to the same conditions as white yeast cells, including elevated temperature (22). Goliath and yeast cells were used to infect oral epithelia and their morphology monitored over time (Fig. 1E and F). Similar to what we had previously observed in a purely *in vitro* system (18), Goliath cells exhibited delayed hyphal germination on oral epithelia in comparison to yeast cells (Fig. 1E and F). However, once hyphae had formed, these were fully capable of invading the host tissue (Fig. 1E).

In order to observe longer-term morphological behavior, individual (~25 per well) cells were incubated on epithelia for 24 h. This approach prevents the extensive overlap of hyphae, which occurs at later time points using conventional inocula and allows morphological analysis using microscopy. Microcolonies formed by the two cell types were visually similar with peripheral hyphae ramifying from a central mycelial mass (Fig. 1G and H). Quantification of diameter revealed that microcolonies formed by Goliath cells were significantly smaller than those formed by yeast cells (Fig. 1G and F), likely due to the observed delay in germination. Nevertheless, when we performed a standard cytotoxicity assay, both yeast and goliath cells caused the same level of epithelial damage (Fig. 1I and J; Fig. S3C).

Therefore, Goliath cells may be more suited for initial colonization of oral tissue due to their enhanced adhesion, and although hyphal germination is delayed, these filaments are able to invade and fully damage oral epithelia. In conclusion, the hyphae formed by Goliath cells appear to be fully pathogenic in this *in vitro* epithelial tissue model.

### Goliath cells are hydrophobic and exhibit preferential adhesion to plastic

**Fig 2:**
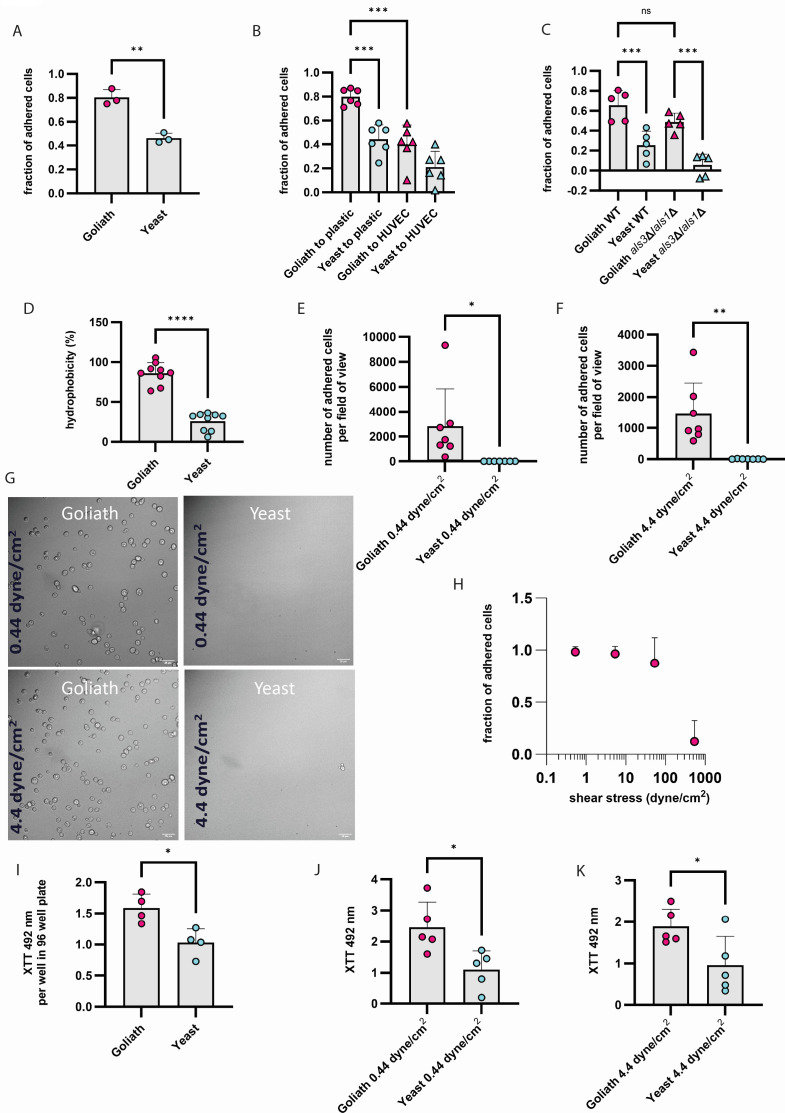
Goliath cell adherence to plastic under physiologically relevant shear stress and biofilm activity. (**A**) Adhesion to plastic. Cells were incubated for 15 min in DMEM media pH 7.4, 5% CO_2_, 37°C. (**B**) Adhesion to plastic and endothelial cells (human umbilical vein endothelial cells, HUVEC). Goliath or yeast cells were incubated in empty wells of 24-well plates and plates with endothelial cell monolayer at the bottom of the wells for 15 min. (**C**) Adhesion to plastic in wild type and *als3*Δ/*als1*Δ double mutant. Cells were incubated for 15 min in empty wells of plastic 24-well plates in DMEM media pH 7.4, 5% CO_2_, 37°C. Wells were then washed three times with PBS, and the percentage adhesion was determined by counting the number of CFU in the supernatant and in the washes. (**D**) Quantification of Goliath and yeast cell wall hydrophobicity, determined by hydrocarbon phase separation assay. (**E–G**) Adhesion to plastic measured by microfluidics. Goliath and yeast cells from 6-day cultures were adjusted to 1 × 10^7^ cells/mL in their own respective culture media (YNB zinc-dropout or YNB zinc-dropout supplemented with 25 µM zinc, respectively). Cells were perfused for 10 min with total volume 2,500 µL (at 0.44 dyne/cm^2^, **E**) or for 1 min with total volume 2,500 µL (at 4.4 dyne/cm^2^, **F**) through ibidi VI channels. Adherence was determined by calculating the average adhered cells on random fields of view. Data from seven independent biological experiments are plotted. (**G**) Example images of cells adherent under shear stress. (**H**) Quantification of the strength of adhesive force of Goliath cells to the plastic surface. Goliath cells were allowed to adhere to the plastic surface for 5 min, and increased shear stress was applied stepwise for 5 min. The number of cells maintaining adherence under increasing shear stress was used to calculate the fraction of adhered cells. (**I, J, K**) Metabolic activity of biofilms seeded by Goliath and yeast cells. (**I**) Biofilms formed from cells seeded without flow. (**J, K**) As above, cells were allowed to adhere under shear stress for 5 min at 0.44 (**J**) or 4.4 dynes/cm^2^ (**K**). Channels were then washed three times with PBS and incubated with RPMI media for 24 h at 37°C in a humidity chamber. Metabolic activity of biofilms seeded by Goliath and yeast cells was then measured by XTT assay. Statistics were performed using unpaired *t*-test. *P < 0.5; **P < 0.01; ***P < 0.001; ****P < 0.0001.

We next examined a potential role for Goliath cells in disseminated candidiasis. As well as increased adhesion to epithelia, Goliath cells were also more adherent to endothelia than yeasts (Fig. 2A).

In the clinic, the presence of an indwelling catheter is one of the major risk factors for systemic candidiasis (5, 9, 10, 12). This is because the catheter can become colonized by fungal cells that form a biofilm. Once formed, the biofilm can release yeast cells (23), seeding the bloodstream and resulting in persistent candidemia (24).

It is thought that *C. albicans* initiates bloodstream infections via yeast translocation from the gastrointestinal tract into the bloodstream (7, 14). This raises the question: How do yeast cells that have translocated from the gut into the bloodstream colonize catheters in the first place?

Goliath cells exhibit higher adhesive potential to abiotic surfaces compared with yeast cells (18). Moreover, Goliath cells adhere better to plastic than to endothelium (Fig. 2B). This may imply that during bloodstream infections, Goliath cells may preferentially attach to indwelling medical devices, over the surrounding endothelium.

We next tested mutants lacking the major known *C. albicans* adhesins: Als1, Als3, Hyr1, and Hwp1 (Fig. 2C; Fig. S3D and E). Each of the mutants formed Goliath cells in response to zinc limitation and, strikingly, exhibited robust adhesion similar with the wild type. Interestingly, yeast cells lacking both *ALS1* and *ALS3* exhibited virtually no adhesion to plastic, but *als1*Δ/*als3*Δ Goliath cells adhered at wild-type levels (Fig. 2C). It would appear, therefore, that the mechanism of Goliath cell adhesion is fundamentally different from those of hyphae. As we could not identify a molecular mechanism of adhesion, we tested a major biophysical property: cell surface hydrophobicity (CSH). The CSH of both yeast and goliath cells was determined using the microbial phase separation assay (25–28). Goliath cells exhibited high hydrophobicity in these assays, which was ~2-fold higher than yeast cells (Fig. 2D). Therefore, CSH may contribute to the enhanced adhesion of *C. albicans* Goliath cells.

### Adhesion under flow is higher for Goliath cells

Circulating *Candida* cells do not encounter indwelling catheters under static conditions as they are inserted within blood vessels.

Shear stress in the circulation is the frictional force generated by blood flowing past surfaces of endothelial cells (or catheter if it is present). It is determined by the mean fluid flow rate, its viscosity, and the physical dimensions of blood vessels (29, 30). All these parameters are highly variable because blood is a non-Newtonian fluid (its viscosity tends to decrease with increasing velocity, and vessels are nonuniform (31). Variations in vessel wall folds and cell structure also create shear stress variations at different points within the same vessel (29). Mean shear stress is lowest in the large veins, which are usually used for catheter insertion, where it is estimated to be less than 1 dyne/cm^2^ (31). We chose shear stress with 0.44 as a low value and 4.4 as a high value. We utilized a microfluidic channel system to emulate the shear stress encountered during blood flow.

When we attempted to colonize abiotic surfaces under physiological flow using our microfluidics platform, we observed no significant adhesion of yeast cells, even at low shear stress of 0.44 dyne/cm^2^ (Fig. 2E and F).

When we flowed in Goliath cells, they attached readily to the perfusion channel. In fact, in pilot experiments, the field of view had become completely occupied with adherent Goliath cells before we took the first image. To investigate how strong the attachment of Goliath cells is, we set up a wash-out microfluidic experiment. Channels were perfused with Goliath cells at 0.5 dyne/cm^2^ for 10 min to allow them to adhere. Shear stresses of 0.5, 5.4, 54, and 540 dyne/cm^2^, representing vena cava, large vein, arteriole, and supra-physiological flow rates, respectively, were then applied. Adherent Goliath cells resisted up to and including 54 dyne/cm^2^, only detaching under supra-physiological shear stress of 540 dyne/cm^2^ (Fig. 2H).

Therefore, Goliath cells appear to be more suited than yeast cells to colonize indwelling medical devices, particularly within the circulatory system.

### Goliath cells pioneer biofilm formation

Adhesion is the first stage of biofilm formation (32, 33). Given the highly adhesive nature of Goliath cells, particularly under conditions of physiological flow, we hypothesized that this novel cell type may be perfectly suited for establishing biofilms.

Biofilm formation is a complex and dynamic process crucial for microbial colonisation of abiotic surfaces, especially medical devices. We used 96-well plastic plates, which were seeded by inoculation with 2 × 10^6^ cells in PBS with shaking at 200 rpm for 90 min at 37°C. Following this incubation, non-adherent cells were washed away with PBS. Then, the cells were incubated in RPMI for 24 h at 37°C under static conditions to allow biofilms to develop. To assess biofilm development, an XTT assay was performed. Under this condition, biofilms formed by Goliath cells exhibited higher XTT activity than those formed by yeast cells (Fig. 2I).

To more closely mimic biofilm formation on indwelling medical devices, ibidi VI channels were perfused with 10^7^ cells/mL Goliath and yeast cells (both in their respective conditioned media) at 0.44 and at 4.4 dyne/cm^2^ for 10 and 1 min, respectively. The channels were flushed with PBS and then incubated in RPMI for 24 h. As expected, the biofilms seeded by Goliath cells were more metabolically active, as assessed by XTT assay (Fig. 2J and K).

We reasoned that the much greater adhesion potential of Goliath cells (Fig. 2A, E and F) may be why Goliath seeded biofilms exhibited such enhanced activity (Fig. 2I through K). To test this, we investigated biofilm architecture. Here, we used comprehensive staining methods that enabled the investigation of Goliath cell seeding behavior, shedding light on the initial stages of biofilm formation. Goliath and yeast cells were seeded to the plastic surfaces with shaking at 200 rpm in ibidi 8-well microscopy slides for 90 min. Following adherence, cells were washed and stained with Concanavalin A–Alexafluor 594 nm. Then, the dye was washed out and the cells incubated to form biofilm for 24 h in RPMI. Following biofilm formation, the entire biofilm was stained with Calcofluor White and 1,778 × 1,780 µm^2^ imaged to a Z depth of 300 µm using a confocal spinning disk microscope. This differential fluorescent staining strategy allowed us to identify those cells, which had initially seeded the biofilm before its formation. It also allowed us to perform 3D rendering of fungal volume of the mature biofilm using Imaris software.

**Fig 3:**
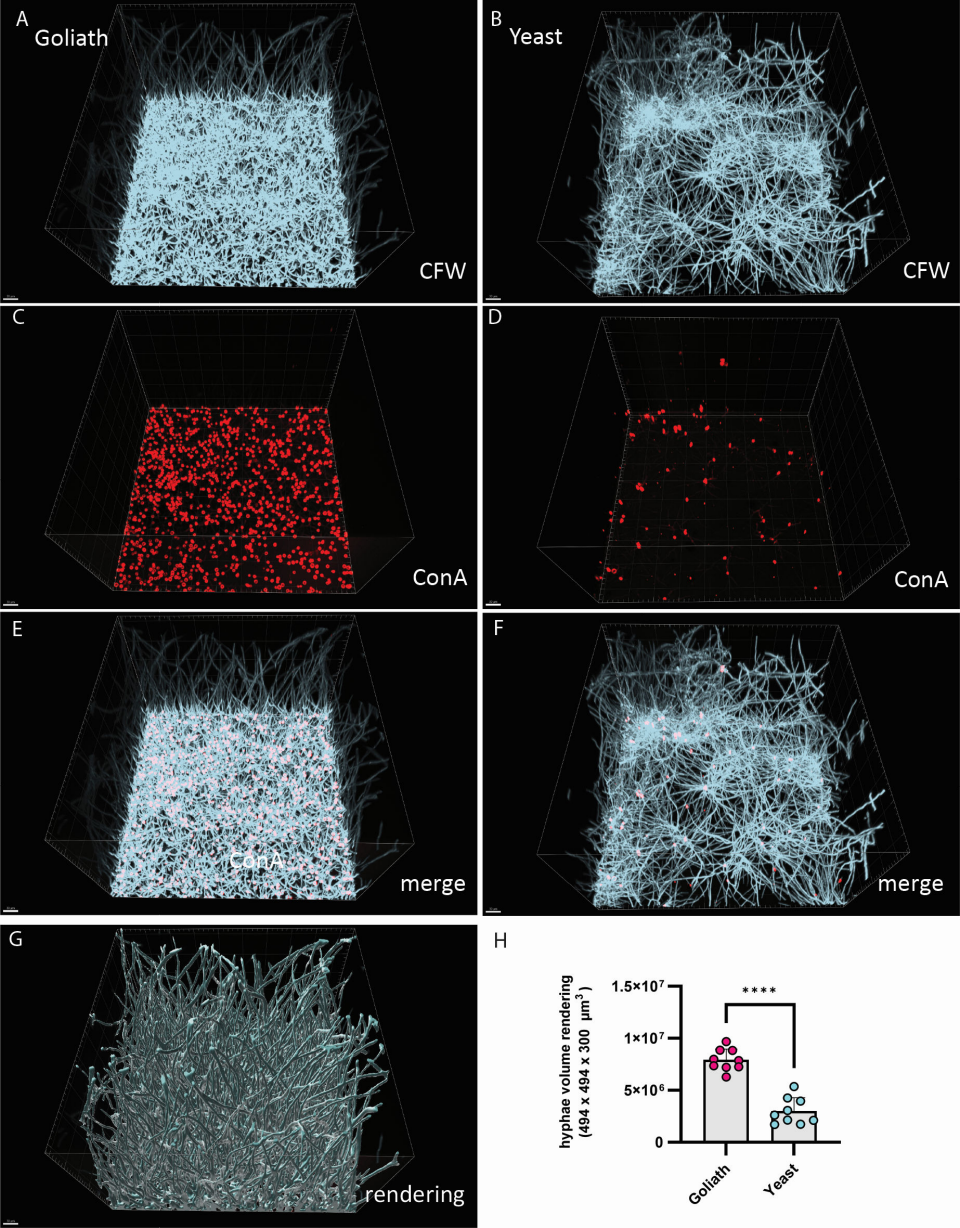
Goliath biofilm architecture. Spinning disc microscopy images of biofilms seeded by Goliath and yeast cells. Indicated cell types were allowed to adhere for 5 min, channels washed three times with PBS and adherent cells stained with Concanavalin A –Alexafluor 594. Channels were then washed and incubated in RPMI at 37°C for 24 h to allow biofilms to form. Biofilms were then stained with Calcofluor White. (A–B) Calcofluor White stained fungal biofilms formed from Goliath and yeast cells. (C–D) Concanavalin A stained initially adherent Goliath and yeast cells. (E–F) Fluorescence overlay of Concanavalin A and Calcofluor White channels. (G) Example of rendering the fungal volume in Calcofluor White channel for the biofilm formed from adherent Goliath cells. Scale bar, 100 µm. (H) Quantification of fungal volume in biofilm rendered using Imaris in Calcofluor White channel. The average hyphal volume of three random fields of view per three independent experiments is plotted. Statistics were performed by unpaired *t*-test. ****P < 0.0001.

**Fig 4:**
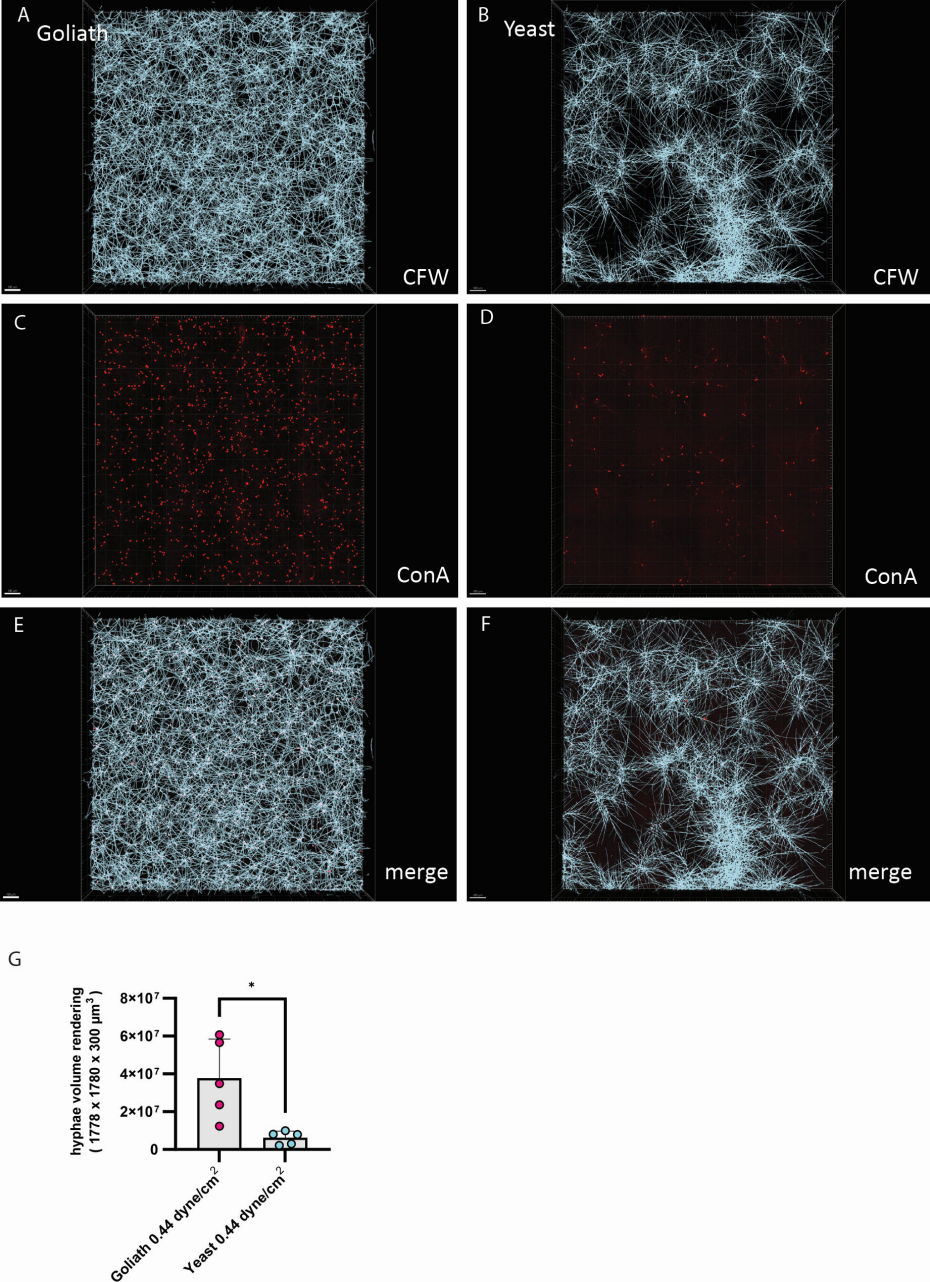
Goliath biofilm architecture in microfluidic channels under shear stress comparable to one in large veins. Goliath and yeast cells were perfused into ibidi VI channels at 0.44 dynes/cm^2^ for 10 min. Channels were washed three times with PBS and adherent cells stained with Concanavalin A –Alexafluor 594. Channels were then washed and incubated in RPMI at 37°C for 24 h to allow biofilms to form. Biofilms were then stained with Calcofluor White. (A–B) Calcofluor White stained fungal biofilms formed from Goliath and yeast cells. (C–D) Concanavalin A stained initially adherent Goliath and yeast cells. (E–F) Fluorescence overlay of Concanavalin A and Calcofluor White channels. (G) Quantification of fungal volume in biofilm rendered using Imaris in Calcofluor White channel. The average hyphal volume of three random fields of view per five independent experiments is plotted. Statistics were performed by unpaired *t*-test. *P < 0.5; ****P < 0.0001.

**Fig 5:**
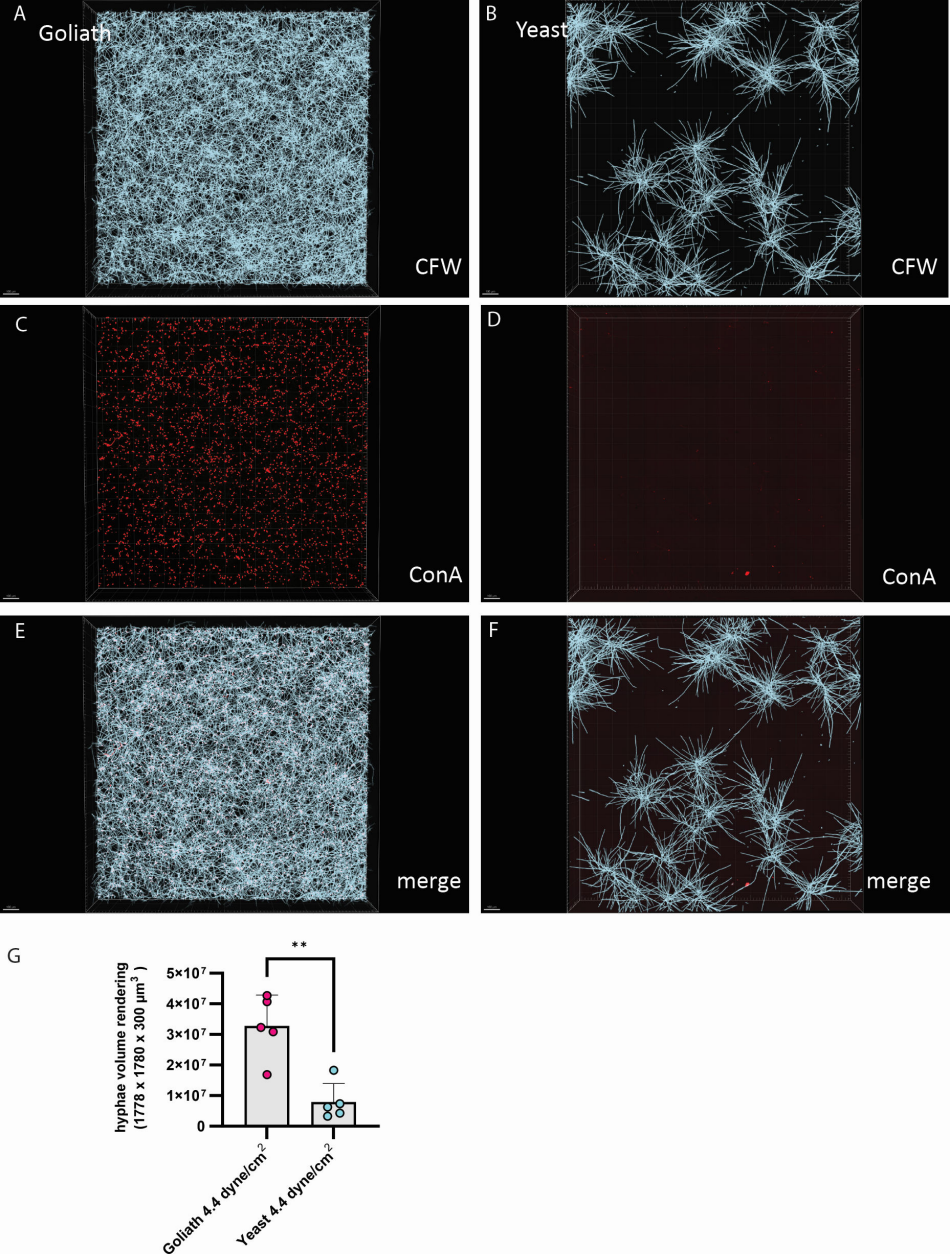
Goliath biofilm architecture in microfluidic channels under shear stress comparable to one in small veins. Goliath and yeast cells were perfused into ibidi VI channels at 4.4 dynes/cm^2^ for 10 min. Channels were washed three times with PBS and adherent cells stained with Concanavalin A –Alexafluor 594. Channels were then washed and incubated in RPMI at 37°C for 24 h to allow biofilms to form. Biofilms were then stained with Calcofluor White. (A–B) Calcofluor White stained fungal biofilms formed from Goliath and yeast cells. (C–D) Concanavalin A stained initially adherent Goliath and yeast cells. (E–F) Fluorescence overlay of Concanavalin A and Calcofluor White channels. (G) Quantification of fungal volume in biofilm rendered using Imaris in Calcofluor White channel. The average hyphal volume of three random fields of view per five independent experiments is plotted. Statistics were performed using unpaired *t*-test. **P < 0.05.

This approach was taken for biofilms seeded with orbital shaking, as well as biofilms seeded by flow at both 0.44 and 4.44 dyne/cm^2^ (Fig. 3 to 5 respectively). In each case, the 24 h RPMI incubated mature biofilm is visualized with Calcofluor White (panels A–B), and the initial seeded Goliath and yeast cells visualised with Concanavalin A - Alexafluor 594 nm (C and D, respectively). The fluorescent overlay of both dyes is displayed (E–F), and the quantification of the 3D rendering (Fig. 3H, 4G and 5G). Additionally, examples of rendered biofilm are shown (Fig. 3G and 5H).

In each case, biofilms seeded with Goliath cells were visually (Fig. 3 to 5A and B) and quantifiably (Fig. 3H, 4G and 5G) denser. In line with our hypothesis, these more robust biofilms initiated by Goliath cells appear to be associated with more effective seeding density (Fig. 3 to 5C and D), very likely due to their enhanced adhesion capacity (Fig. 2A, E and F).

In summary, Goliath cells appear to be very well suited to pioneer the colonization of abiotic surfaces, particularly under conditions of physiological shear stress.

## DISCUSSION

*C. albicans* demonstrates remarkable morphological plasticity in response to environmental cues, switching between multiple morphotypes, which can contribute towards both commensalism and pathogenic growth. While various morphological types exist (34), most research is focused on yeast and hyphae. Goliath cells are a newly identified morphological variant of *C. albicans*, induced by zinc limitation (18).

We currently have limited knowledge of Goliath cell physiology, specifically whether they may contribute toward commensalism or pathogenicity. Our study therefore focused on their pathogenic potential and medical relevance.

We are unaware of previous descriptions of large *C. albicans* yeast cells *in vivo*. This is perhaps not surprising. Unlike Cryptococcal Titan cells, which can enlarge up to 100 µm in diameter and are readily identified *in vivo* (35), *Candida* Goliath cells are, on average, only four times larger by volume than regular yeasts (18). It is possible that this more subtle size difference may have been overlooked by histological analysis.

On host tissue, Goliath cells germinated hyphae, which invaded the oral epithelium. Despite a delay in hyphal germination, they ultimately cause similar levels of damage as hyphae produced by yeast cells. From these experiments, we conclude that Goliath cells have similar pathogenic potential to yeast cells.

The most striking phenotype of Goliath cells compared with normal yeasts is their hyper-adhesiveness to plastic surfaces (18). In the current study, we built on this discovery.

Goliath cells adhered more effectively to oral and vaginal epithelial cells. They were also more adherent to enterocytes and endothelia, but this was not significant. This suggests that Goliath cells may have a general advantage over yeast cells at colonizing host tissue. However, we discovered that Goliath cells prefer to attach to plastic compared to host tissue.

It is well documented that the *C. albicans* yeast-to-hypha transition facilitates robust adhesion to both biotic and abiotic surfaces. This is because hyphae, but not yeast cells, express several adhesin proteins on their cell surface. The most important of these are Als1/3, Hwp1, and Hyr1. However, Goliath cells lacking *ALS1* and *ALS3*, *HWP1*, or *HYR1* exhibited wild-type adhesion. In fact, the adhesion capacity of the *als1*Δ/*als3*Δ double mutant rose considerably following the yeast-to-Goliath switch. Together, this suggests that the adhesion mechanism of Goliath cells is fundamentally different from that of adhesin-expressing hyphae. Increased cell surface hydrophobicity may contribute to adhesion. Interestingly, this biophysical property has recently been shown to mediate *Candida auris* adhesion (36).

In the case of bloodstream infections caused by *Candida parapsilosis* and, more recently, *Candida auris*, it is possible that catheter contamination could occur directly at the port of entry. This is because these species colonize the human skin.

On the other hand, it is thought that systemic *C. albicans* infections initiate when the fungus translocates from the gastrointestinal tract to the bloodstream and that it is yeast cells that disseminate in the circulation (14). In this context, biofilms formed on indwelling medical devices, such as catheters, must be first colonized by circulating cells.

It is possible that some yeast cells will undergo hyphal morphogenesis, permitting colonization and subsequent biofilm formation. However, human blood represses complete hypha formation (37) and, in a mouse model of disseminated candidiasis, fungal cells are rapidly cleared from the bloodstream. In fact, MacCallum and Odds observed one log_10_ clearance of circulating *C. albicans* cells within 10 min of intravenous injection (38).

These observations suggest that circulating yeast cells would not be well-poised to colonise indwelling medical devices from the bloodstream.

In the current study, we simulated this physical aspect of the bloodstream by applying shear stress.

We observed minimal adhesion of yeast cells to plastic surfaces, even at low flow rates. Goliath cells, on the other hand, adhered robustly to abiotic surfaces, maintaining attachment even under high-shear stress conditions. We propose that Goliath cells are better suited than yeasts for colonizing indwelling medical devices, such as catheters, from the bloodstream.

To complement standard XTT assays, we developed advanced spinning disc microscopy, differential staining, and refractive index adjustment to study biofilm structure. Our differential staining approach revealed a much denser layer of initially adherent Goliath cells compared to yeasts. This basal layer germinated vertical hyphae, which aligned parallel with each other, forming a much denser biofilm than observed for those formed by adherent yeast cells.

Understanding the mechanisms behind this phenomenon opens avenues for strategies in catheter surface modifications, offering potential solutions for addressing biofilm-related complications in various medical applications.

In summary, this study identifies a medically important property of Goliath cells – this morphotype is well-suited to colonize and form biofilms on indwelling medical devices from the circulation.

## MATERIALS AND METHODS

### Strain and culture conditions

*C. albicans* wild-type laboratory strain DAY185 (ura3Δ::imm434HIS1::his1::hisGARG4::URA3::arg4::hisG ura3Δ::λimm434his1::hisGarg4::hisG) was maintained on YPD agar (1% yeast extract (Formedium, UK), 2% mycological peptone (Formedium, UK), 2% D-glucose, and 2% agar (Formedium, UK). Liquid overnight cultures were grown in 2% glucose, 2% YNB supplemented with NH_4_SO_4_ medium (Formedium, UK) in a shaking incubator at 30°C and 200 rpm. The mutant strains (CJN1348, als1/als1 als3/als3 double mutant (39), CJN1352, als1/als1 als3/als3::ALS1 prototrophic strain, bCJN1356, als1/als1 als3/als3::ALS3 prototrophic strain [40]) were kindly provided by Prof. Aaron Mitchell. Null mutant for HYR1 (URA blaster), transformed with CIp10-HYR1 containing the ORF + 1 kb up/down is from Rodrigo Belmonte (41). Strain CAH7-1A1E2 hwp1Δ/hwp1Δ hwp1::hisG/hwp1Δ::hisG eno1::URA3 (URA3 reintegrated at the ENO1 locus) (42) was provided by Janet Staab, Columbus, OH, USA.

### Goliath cell induction

Zinc-depleted conditions were achieved by washing cells from an SD preculture in 1 mM EDTA, then three times with ultrapure water, and inoculating at an OD600 0.05 to YNB Zn dropout 2% glucose (Formedium, UK). The cells were incubated in a shaking incubator at 200 rpm, 30°C, for 6 days. We used cells grown in YNB Zn dropout 2% glucose (Formedium, UK) supplemented with 25 µM ZnSO4 as a control.

### Microfluidics

The chip was designed in AutoCAD software. The trap dimensions are the following: the distance between pillars in the trap for mother cell is 6.75 µm, the distance in the chamber to collect the progeny is 25 µm, the length of the pillars is 68 µm, height of the chip 11 µm. The full chip design is shown in Fig. S1. The photoresist SU-8 mold was ordered from Microresist (Germany). Chips were produced by standard soft lithography using PDMS kit (Ellsworth, UK). Chips were bound to coverslips using a plasma machine (Zepto, Diener electronic, Germany). Chips were mounted on a DeltaVision Elite microscope (IMSOL, UK), which was equipped with a heated chamber (30°C). The chip was connected by PTFE tubing (Adtech, UK) to 10 mL syringes and perfused with YNB using motorised syringe pumps (World Precision Instruments, Sarasota, Florida, www.wpiinc.com) with a speed of 1 µL/min. Images were taken every hour with DeltaVision software.

### Pulse labeling with FITC

Live *C. albicans* cells were stained for 10 min at room temperature in the dark with 1 mg/mL fluorescein isothiocyanate (FITC) (Sigma, United Kingdom) in 0.05 M carbonate-bicarbonate buffer (pH 9.6) (BDH Chemicals, VWR International, UK). Following incubation, cells were washed three times in 1 × PBS to remove any residual FITC and finally resuspended in 1 × PBS. FITC-stained yeast were inoculated into 4 mL of YNB (Formedium, UK). Every 24 h, 10 µL samples were taken for imaging with DeltaVision Elite microscope (IMSOL, UK) and linear measurement of long and short axes was taken in ImageJ. Volume of cells was calculated in MATLAB using formula for ellipsoid volume $V=\frac{4}{3}\pi *a*b*c$, where *a*, *b*, and *c* stand for axes of ellipsoid.

### Adhesion assays

#### Static adhesion

Goliath cells and yeast control were washed in PBS and diluted in DMEM (Gibco) to 10^7^ cells/mL. 300 µL of cells in DMEM were placed per well of 24-well plate (Costar by Corning, Fisher, UK) at 37°C CO^2^ 5% for 15 min. Following incubation, supernatant from wells was collected. The wells were then washed three times with PBS. Washes from each well were also collected. Supernatant and washes were then appropriately diluted and plated onto YPD agar. CFUs obtained following incubation were counted with ImageJ to determine the percentage of detached and adherent cells.

#### Flow adhesion

A microfluidic system was employed to simulate blood flow shear stress conditions. Yeast and Goliath cells were pumped (syringe pump from World Precision Instruments, Sarasota, Florida, https://www.wpiinc.com/) into the channels, and adhesion dynamics were observed under flow rates, ranging from low to high shear stress. Goliath cells and yeast were diluted to 10^7^ cells/mL in their respective supernatant. The ibidi 8-well chip was perfused using pumps for 10 min at a rate of 250 µL/min and 1 min at a rate of 2,500 µL/min. Shear stress was calculated according to ibidi channel dimensions and instructions. According to the manufacturer, slides were made of polyethylene derivative with ibiTreat surface for cell culture. Images were taken during adhesion using the DeltaVision Elite microscope (IMSOL, UK). Channels were washed with PBS three times, and images of neighbouring fields were taken at 40× magnification.

#### Wash-out assay

Following cellular attachment, Goliath and yeast cells were subjected to increasing shear stress in a controlled flow system. Adhesion strength and persistence were assessed under different shear stress conditions to mimic physiological blood flow. The fraction of cells maintaining adhesion was calculated.

### Hydrophobicity assay

Cell surface hydrophobicity (CSH) was assessed using the microbial adhesion assay to hydrocarbons (25–28). Goliath cells and yeast were harvested and washed with PBS. A cell suspension of OD_600_ nm 0.5 was prepared in PBS (A0); 3 mL of this yeast suspension was overlaid with 0.4 mL of the hydrophobic hydrocarbon n-octane (Sigma-Aldrich). After vigorous vortexing, phases were allowed to separate for 5 min at 22°C and the OD_600_ nm of the aqueous phase was measured (A1). The percentage of hydrophobicity was calculated as follows: hydrophobicity CSH(%) = [1−(A1/A0)] ×100. All assays represent at least six independent experiments performed in triplicate.

### Biofilm formation and staining

#### Rotational shaking biofilm seeding

Goliath cells and yeast were diluted in PBS to 1 × 10^7^ cells/mL in PBS. Cell suspension in PBS (400 µl per well) was placed in Ibidi 8-well (Ibidi) and incubated for 90 min while shaking at 200 rpm, 37°. Biofilms were grown in RPMI for 24 hrs, 37°. Concanavalin A - Alexa Fluor 594 nm was used for staining initially attached cells for 15 min. Then, after PBS wash, the biofilm was left to grow in RPMI for 24 hrs at 37°C. After 24 h, the biofilm was fixed in 4% PFA, washed in PBS, and stained with Calcofluor White.

#### Flow-seeded biofilm

Goliath cells and yeast were injected into an ibidi VI 0.4 mm high chip with shear stress 0.44 and 4.4 dyne/cm^2^ for 10 and 1 min, respectively. The channels were washed with PBS, stained with Concanavalin A-AlexaFluor 594 nm for 15 min, washed with PBS, and filled with RPMI. Chips were incubated at 37°C for 24 h to form biofilm.

#### Biofilm in 96-well plate for XTT assay

Goliath cells and yeast were diluted in PBS to 1 × 10^7^ cells/mL. Then, 200 µl per well of cells in PBS was placed in a 96-well plate (Costar by Corning, Fisher, UK) and incubated for 90 min while shaking at 200 rpm, 37°C. Biofilms were then allowed to develop in RPMI at 37°C for 24 h.

### Imaging

Morphological characterisation of single colonies grown on top of oral epithelium monolayers, cell volume measurements, germination of hyphae, and invasion host tissues were imaged using DeltaVision Elite microscope (IMSOL, UK) (DAPI and FITC filter cubes). Biofilms were imaged using a confocal spinning disk microscope Dragonfly 505 (Andor, Oxford Instruments, UK). Z-stack was set up to have 1 µm step between optical sections, and 300 µm volume was acquired. For biofilm formed in 8-well slides (ibidi, Germany), three random fields were imaged per independent experiment. For biofilms formed in ibidi VI 0.4 mm channels, 4 × 4 fields were imaged per independent experiment and stitched with Imaris software. Lens x25 was used. Refractive index adjustment was achieved with 33% iohexol (Sigma, UK) in TRIS-EDTA buffer pH 7.4. Rendering of hyphal volume was performed with Imaris software.

### Cell culture and infection

To assess the capacity of Goliath cells to form hyphae, Goliath cells and yeast were washed in PBS, and 200 µL of 10^7^ cells were inoculated in DMEM (Gibco, Thermo Fisher Scientific, UK) in a 96-well plate. Plates were incubated at 37°C and 5% CO2 for 90, 180, or 360 min, fixed and stained with Concanavalin A - Alexafluor 594 nm, permeabilized with 0.1% Triton, and then stained with Calcofluor White. The XTT assay (CyQUANT XTT Cell Viability Assay, Invitrogen) assessed candida metabolic activity in biofilms. We followed the manufacturer’s instructions.

### Statistical analysis

Statistical analyses were conducted using GraphPad Prism 5, employing student *t*-test and ANOVA. All experiments were performed at least three times independently. Results are presented as mean values with standard deviations, with *P*-values indicating significance. Graphs and figures were generated using the statistical software GraphPad Prism, and representative images were chosen to convey key findings visually.

## SUPPLEMENTAL MATERIAL

Figure S1 mbio.03425-24-s0001.tif Microfluidic chip design.

Figure S2 mbio.03425-24-s0002.tif Goliath cell formation.

Figure S3 mbio.03425-24-s0003.tif Goliath cell adherence.

Supplemental Legends mbio.03425-24-s0004.docx Legends for Fig. S1 to S3.

ASM does not own the copyrights to Supplemental Material that may be linked to, or accessed through, an article. The authors have granted ASM a non-exclusive, world-wide license to publish the Supplemental Material files. Please contact the corresponding author directly for reuse.

## References

[1] Brown GD, Ballou ER, Bates S, Bignell EM, Borman AM, Brand AC, Brown AJP, Coelho C, Cook PC, Farrer RA, Govender NP, Gow NAR, Hope W, Hoving JC, Dangarembizi R, Harrison TS, Johnson EM, Mukaremera L, Ramsdale M, Thornton CR, Usher J, Warris A, Wilson D. 2024. The pathobiology of human fungal infections. Nat Rev Microbiol 22:687–704. doi:10.1038/s41579-024-01062-w 38918447

[2] Jacobsen ID, Hube B. 2017. Candida albicans morphology: still in focus. Expert Rev Anti Infect Ther 15:327–330. doi:10.1080/14787210.2017.1290524 28152317

[3] Kumar D, Kumar A. 2024. Molecular determinants involved in Candida albicans biofilm formation and regulation. Mol Biotechnol 66:1640–1659. doi:10.1007/s12033-023-00796-x 37410258

[4] Bassetti M, Giacobbe DR, Vena A, Trucchi C, Ansaldi F, Antonelli M, Adamkova V, Alicino C, Almyroudi M-P, Atchade E, et al.. 2019. Incidence and outcome of invasive candidiasis in intensive care units (ICUs) in Europe: results of the EUCANDICU project. Crit Care 23:219. doi:10.1186/s13054-019-2497-3 31200780 PMC6567430

[5] Klingspor HT, Hällgren A. 2024. Factors influencing outcomes in candidemia: a retrospective study of patients in a Swedish county. Mycoses 67:e13758. doi:10.1111/myc.13758 38932675

[6] Lass-Flörl C, Kanj SS, Govender NP, Thompson GR 3rd, Ostrosky-Zeichner L, Govrins MA. 2024. Invasive candidiasis. Nat Rev Dis Primers 10:20. doi:10.1038/s41572-024-00503-3 38514673

[7] Sprague JL, Schille TB, Allert S, Trümper V, Lier A, Großmann P, Priest EL, Tsavou A, Panagiotou G, Naglik JR, Wilson D, Schäuble S, Kasper L, Hube B. 2024. Candida albicans translocation through the intestinal epithelial barrier is promoted by fungal zinc acquisition and limited by NFκB-mediated barrier protection. PLoS Pathog 20:e1012031. doi:10.1371/journal.ppat.1012031 38427950 PMC10907035

[8] Jung P, Mischo CE, Gunaratnam G, Spengler C, Becker SL, Hube B, Jacobs K, Bischoff M. 2020. Candida albicans adhesion to central venous catheters: Impact of blood plasma-driven germ tube formation and pathogen-derived adhesins. Virulence 11:1453–1465. doi:10.1080/21505594.2020.1836902 33108253 PMC7595616

[9] Díaz-Navarro M, Samaniego R, Piqueras JC, Díez R, Hafian R, Manzano I, Muñoz P, Guembe M. 2023. Understanding the diagnosis of catheter-related bloodstream infection: real-time monitoring of biofilm growth dynamics using time-lapse optical microscopy. Front Cell Infect Microbiol 13:1286527. doi:10.3389/fcimb.2023.1286527 38125909 PMC10731284

[10] Hashemi Fesharaki S, Aghili SR, Shokohi T, Boroumand MA. 2018. Catheter-related candidemia and identification of causative Candida species in patients with cardiovascular disorder. Curr Med Mycol 4:7–13. doi:10.18502/cmm.4.2.63 PMC6181067 30324151

[11] Meawed TE, AlNakeera AM, Attia O, Hassan NAM, Anis RH. 2024. Candida auris central line-associated blood stream infection in critically ill patients: the worst end of a bad scenario. Int Microbiol 28:377–383. doi:10.1007/s10123-024-00545-3 38940863

[12] Pitiriga VC, Bakalis J, Campos E, Kanellopoulos P, Sagris K, Saroglou G, Tsakris A. 2024. Central venous catheters versus peripherally inserted central catheters: a comparison of indwelling time resulting in colonization by multidrug-resistant pathogens. Antibiotics (Basel) 13:89. doi:10.3390/antibiotics13010089 38247648 PMC10812679

[13] Wijaya M, Halleyantoro R, Kalumpiu JF. 2023. Biofilm: the invisible culprit in catheter-induced candidemia. AIMS Microbiol 9:467–485. doi:10.3934/microbiol.2023025 37649801 PMC10462453

[14] Sprague JL, Kasper L, Hube B. 2022. From intestinal colonization to systemic infections: Candida albicans translocation and dissemination. Gut Microbes 14:2154548. doi:10.1080/19490976.2022.2154548 36503341 PMC9746630

[15] Katsipoulaki M, Stappers MHT, Malavia-Jones D, Brunke S, Hube B, Gow NAR. 2024. Candida albicans and Candida glabrata: global priority pathogens. Microbiol Mol Biol Rev 88:e0002123. doi:10.1128/mmbr.00021-23 38832801 PMC11332356

[16] Wilson D, Deepe GS. 2019. The intersection of host and fungus through the zinc lens. Curr Opin Microbiol 52:35–40. doi:10.1016/j.mib.2019.04.008 31132743 PMC6874917

[17] Wilson D. 2021. The role of zinc in the pathogenicity of human fungal pathogens. Adv Appl Microbiol 117:35–61. doi:10.1016/bs.aambs.2021.09.001 34742366

[18] Malavia D, Lehtovirta-Morley LE, Alamir O, Weiß E, Gow NAR, Hube B, Wilson D. 2017. Zinc limitation induces a hyper-adherent goliath phenotype in Candida albicans. Front Microbiol 8:2238. doi:10.3389/fmicb.2017.02238 29184547 PMC5694484

[19] Crawford A, Wilson D. 2015. Essential metals at the host-pathogen interface: nutritional immunity and micronutrient assimilation by human fungal pathogens. FEMS Yeast Res 15:fov071. doi:10.1093/femsyr/fov071 26242402 PMC4629794

[20] Roselletti E, Pericolini E, Nore A, Takacs P, Kozma B, Sala A, De Seta F, Comar M, Usher J, Brown GD, Wilson D. 2023. Zinc prevents vaginal candidiasis by inhibiting expression of an inflammatory fungal protein. Sci Transl Med 15:eadi3363. doi:10.1126/scitranslmed.adi3363 38055800 PMC7616067

[21] Wächtler B, Wilson D, Haedicke K, Dalle F, Hube B. 2011. From attachment to damage: defined genes of Candida albicans mediate adhesion, invasion and damage during interaction with oral epithelial cells. PLoS One 6:e17046. doi:10.1371/journal.pone.0017046 21407800 PMC3044159

[22] Si H, Hernday AD, Hirakawa MP, Johnson AD, Bennett RJ. 2013. Candida albicans white and opaque cells undergo distinct programs of filamentous growth. PLoS Pathog 9:e1003210. doi:10.1371/journal.ppat.1003210 23505370 PMC3591317

[23] Uppuluri P, Chaturvedi AK, Srinivasan A, Banerjee M, Ramasubramaniam AK, Köhler JR, Kadosh D, Lopez-Ribot JL. 2010. Dispersion as an important step in the Candida albicans biofilm developmental cycle. PLoS Pathog 6:e1000828. doi:10.1371/journal.ppat.1000828 20360962 PMC2847914

[24] Kullberg BJ, Arendrup MC. 2015. Invasive candidiasis. N Engl J Med 373:1445–1456. doi:10.1056/NEJMra1315399 26444731

[25] Danchik C, Casadevall A. 2020. Role of cell surface hydrophobicity in the pathogenesis of medically-significant fungi. Front Cell Infect Microbiol 10:594973. doi:10.3389/fcimb.2020.594973 33569354 PMC7868426

[26] Salas-Tovar JA, Escobedo-García S, Olivas GI, Acosta-Muñiz CH, Harte F, Sepulveda DR. 2021. Method-induced variation in the bacterial cell surface hydrophobicity MATH test. J Microbiol Methods 185:106234. doi:10.1016/j.mimet.2021.106234 33971217

[27] Straver MH, Kijne JW. 1996. A rapid and selective assay for measuring cell surface hydrophobicity of brewer’s yeast cells. Yeast 12:207–213. doi:10.1002/(SICI)1097-0061(19960315)12:3<207::AID-YEA899>3.0.CO;2-U 8904332

[28] Vij R, Danchik C, Crawford C, Dragotakes Q, Casadevall A. 2020. Variation in cell surface hydrophobicity among Cryptococcus neoformans strains influences Interactions with amoebas. mSphere 5:e00310-20. doi:10.1128/mSphere.00310-20 32350094 PMC7193044

[29] Lu D, Kassab GS. 2011. Role of shear stress and stretch in vascular mechanobiology. J R Soc Interface 8:1379–1385. doi:10.1098/rsif.2011.0177 21733876 PMC3163429

[30] Paszkowiak JJ, Dardik A. 2003. Arterial wall shear stress: observations from the bench to the bedside. Vasc Endovascular Surg 37:47–57. doi:10.1177/153857440303700107 12577139

[31] Ballermann BJ, Dardik A, Eng E, Liu A. 1998. Shear stress and the endothelium. Kidney Int 54:S100–S108. doi:10.1046/j.1523-1755.1998.06720.x 9736263

[32] Blankenship JR, Mitchell AP. 2006. How to build a biofilm: a fungal perspective. Curr Opin Microbiol 9:588–594. doi:10.1016/j.mib.2006.10.003 17055772

[33] de Barros PP, Rossoni RD, de Souza CM, Scorzoni L, Fenley JDC, Junqueira JC. 2020. Candida biofilms: an update on developmental mechanisms and therapeutic challenges. Mycopathologia 185:415–424. doi:10.1007/s11046-020-00445-w 32277380

[34] Gow NAR, Yadav B. 2017. Microbe Profile: Candida albicans: a shape-changing, opportunistic pathogenic fungus of humans. Microbiology (Reading, Engl) 163:1145–1147. doi:10.1099/mic.0.000499 28809155

[35] Dambuza IM, Drake T, Chapuis A, Zhou X, Correia J, Taylor-Smith L, LeGrave N, Rasmussen T, Fisher MC, Bicanic T, Harrison TS, Jaspars M, May RC, Brown GD, Yuecel R, MacCallum DM, Ballou ER. 2018. The Cryptococcus neoformans Titan cell is an inducible and regulated morphotype underlying pathogenesis. PLoS Pathog 14:e1006978. doi:10.1371/journal.ppat.1006978 29775474 PMC5959070

[36] Santana DJ, Anku JAE, Zhao G, Zarnowski R, Johnson CJ, Hautau H. 2023. A Candida auris-specific adhesin, Scf1, governs surface association, colonization, and virulence. Science 381:1461–1467. doi:10.1126/science.adf8972 37769084 PMC11235122

[37] Fradin C, De Groot P, MacCallum D, Schaller M, Klis F, Odds FC, Hube B. 2005. Granulocytes govern the transcriptional response, morphology and proliferation of Candida albicans in human blood. Mol Microbiol 56:397–415. doi:10.1111/j.1365-2958.2005.04557.x 15813733

[38] MacCallum DM, Odds FC. 2005. Temporal events in the intravenous challenge model for experimental Candida albicans infections in female mice. Mycoses 48:151–161. doi:10.1111/j.1439-0507.2005.01121.x 15842329

[39] Nobile CJ, Andes DR, Nett JE, Smith FJ, Yue F, Phan Q-T, Edwards JE, Filler SG, Mitchell AP. 2006. Critical role of Bcr1-dependent adhesins in C. albicans biofilm formation in vitro and in vivo. PLoS Pathog 2:e63. doi:10.1371/journal.ppat.0020063 16839200 PMC1487173

[40] Nobile CJ, Schneider HA, Nett JE, Sheppard DC, Filler SG, Andes DR, Mitchell AP. 2008. Complementary adhesin function in C. albicans biofilm formation. Curr Biol 18:1017–1024. doi:10.1016/j.cub.2008.06.034 18635358 PMC2504253

[41] Rudkin FM, Raziunaite I, Workman H, Essono S, Belmonte R, MacCallum DM, Johnson EM, Silva LM, Palma AS, Feizi T, Jensen A, Erwig LP, Gow NAR. 2018. Single human B cell-derived monoclonal anti-Candida antibodies enhance phagocytosis and protect against disseminated candidiasis. Nat Commun 9:5288. doi:10.1038/s41467-018-07738-1 30538246 PMC6290022

[42] Sundstrom P, Cutler JE, Staab JF. 2002. Reevaluation of the role of HWP1 in systemic candidiasis by use of Candida albicans strains with selectable marker URA3 targeted to the ENO1 locus. Infect Immun 70:3281–3283. doi:10.1128/IAI.70.6.3281-3283.2002 12011025 PMC128023

