# Supplementary figures and images for: Candida albicans Goliath cells pioneer biofilm formation
Source: mBio. 2025 Aug 8;16(9):e03425-24. doi: 10.1128/mbio.03425-24 (PMC12421812; doi:10.1128/mbio.03425-24)

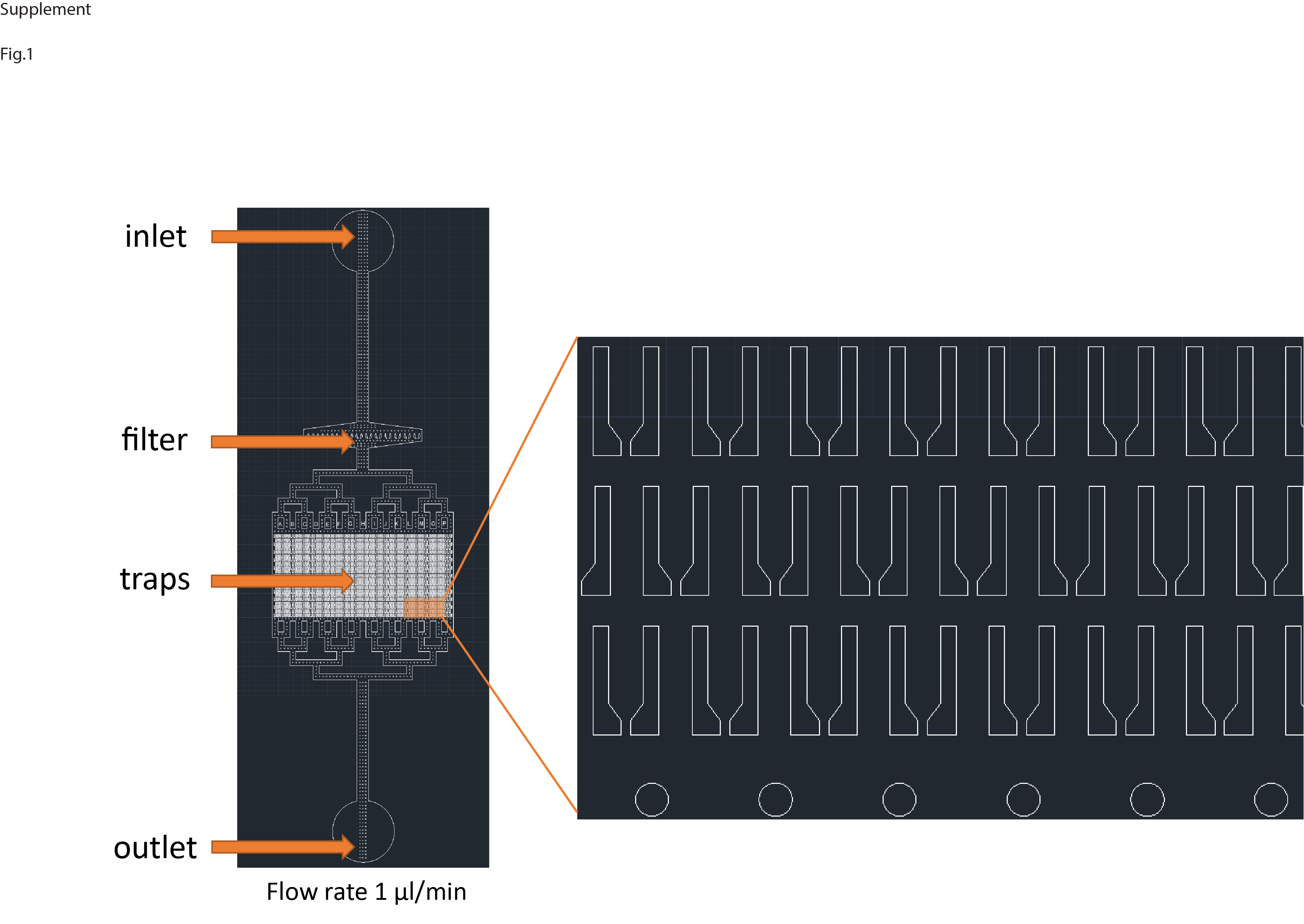

Supplement: Figure S1 — Microfluidic chip design. [file mbio.03425-24-s0001.tif]

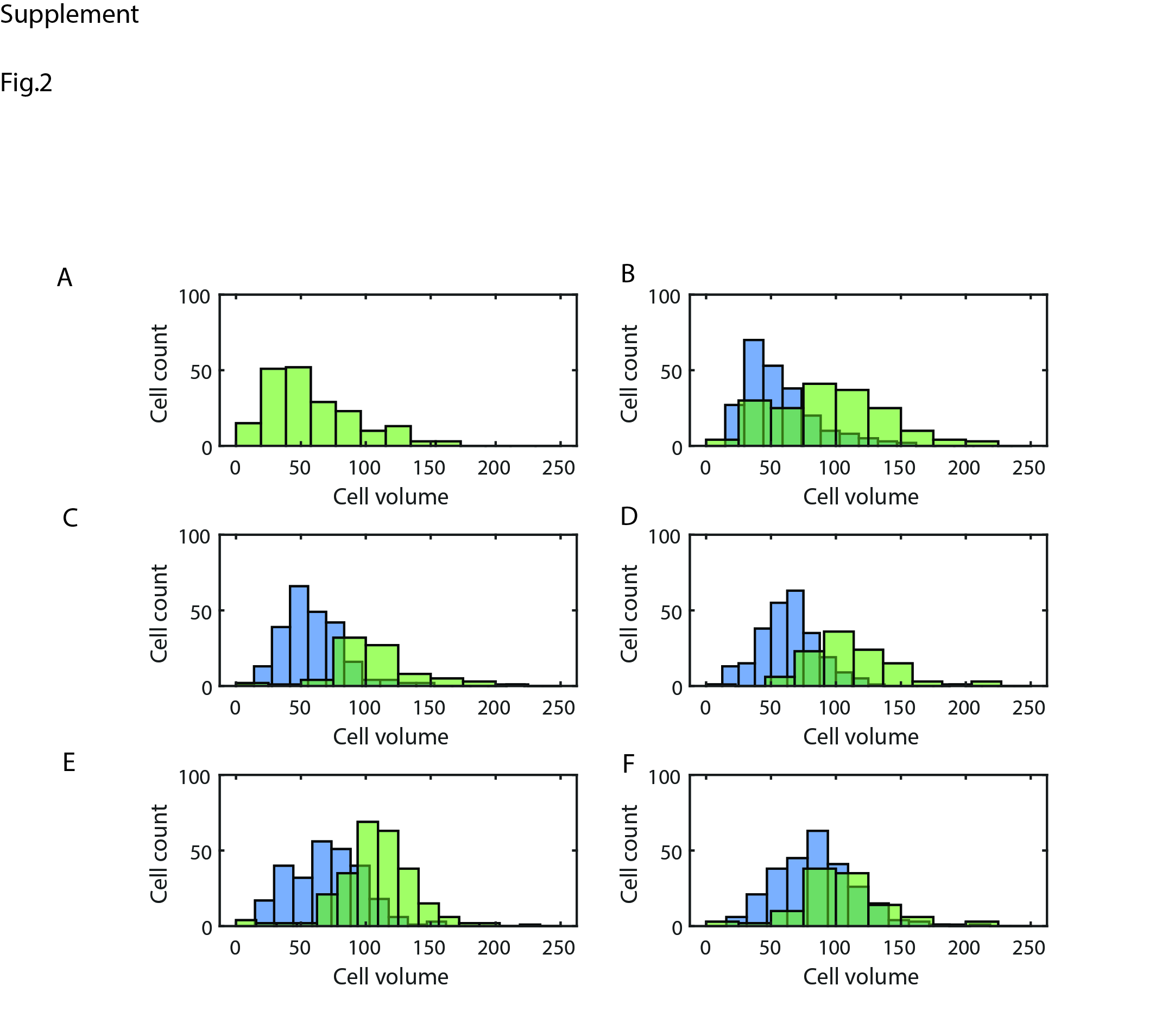

Supplement: Figure S2 — Goliath cell formation. [file mbio.03425-24-s0002.tif]

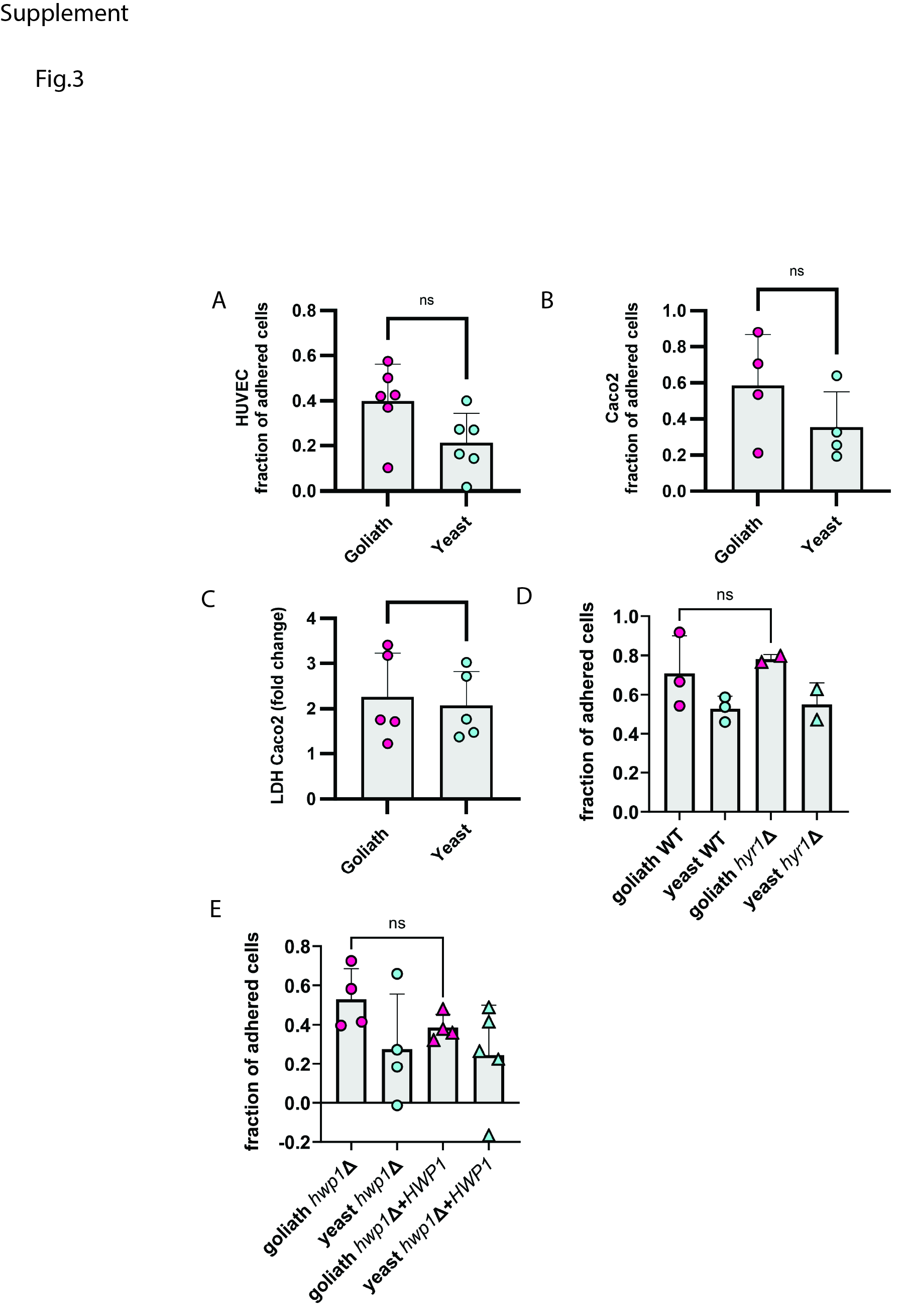

Supplement: Figure S3 — Goliath cell adherence. [file mbio.03425-24-s0003.tif]
