# Supplementary material for: Candida albicans Goliath cells pioneer biofilm formation
Source: mBio. 2025 Aug 8;16(9):e03425-24. doi: 10.1128/mbio.03425-24 (PMC12421812; doi:10.1128/mbio.03425-24)
Supplement: Supplemental Legends — Legends for Fig. S1 to S3. [file mbio.03425-24-s0004.docx]

**Supplementary Fig.1 Microfluidic chip design.**

Array of hydrodynamical traps designed to collect Goliath cells progeny. Single trap has dimensions: of 25 µm x 68 µm x 11 µm with pillars spaced 6.75 µm apart from each other at the bottle neck point.

**Supplementary Fig.2 Goliath cells formation.**

(**A**) Histogram of yeast cell volumes stained with FITC (green) before inoculation into YNB without zinc and incubated for 6 days. (**B**-**F**) Volumes of emergent Goliath cells (green) and unstained daughter cells (blue) (**B**, 24 h; **C**, 48 h; **D**, 72 h; **E**, 96 h; **F**, 120 h post-inoculation).

**Supplementary Fig 3.** **Goliath cell's adherence.**

Goliath or yeast cells were incubated on tissue culture monolayer for 15 minutes. Endothelial or epithelia were then washed three times with PBS, and the percentage adhesion was determined by counting the number of CFU in the supernatant and in the washes. (**A**) Adhesion of Goliath cells and yeast to endothelial cells (HUVEC). (**B**) Adhesion of Goliath cells and yeast to Caco2 cell monolayer. (**C**) Caco2 epithelial damage measured by LDH assay after 24 hrs incubation. (**D**) Adhesion to plastic of wild type and *hyr1*Δ Goliath and yeast cells. (**E**) Adhesion to plastic of wild type and *hwp1*Δ Goliath and yeast cells
